# Tcf12 balances the reconstitution and differentiation capacity of hematopoietic stem cell

**DOI:** 10.1097/BS9.0000000000000059

**Published:** 2020-12-01

**Authors:** Min Liao, Jianwei Wang

**Affiliations:** School of Pharmaceutical Sciences, Tsinghua University, Beijing 100084, China

**Keywords:** Hematopoietic stem cell, Proliferation, Tcf12

## Abstract

Tcf12 has been identified as one of the main helix-loop-helix transcription factors that regulates T cell development from double negative to double positive stage transition. While, the function of Tcf12 in hematopoietic stem cells remains not investigated. In this study, we observed that Tcf12 is expressed in HSCs and targeted deletion of Tcf12 in hematopoietic cells results in increased frequency and absolute number of HSCs, but compromises the reconstitution capacity of HSCs. Further analysis reveals that Tcf12 is dispensable for the self-renewal of HSCs. The declined reconstituted capacity of Tcf12^−/−^ HSCs stems from the decrease in the ability to differentiate into lymphoid-primed multipotent progenitors, and furthermore B and T lineages.

## 1 INTRODUCTION

Tcf12 (transcription factor 12), one of the basic helix-loop-helix (bHLH) transcription factors, is differentially expressed at different stages of hematopoiesis.^1,2^ It has been shown that cellular differentiation is well regulated by proteins with the basic-helix-loop-helix domain.^3^ The bHLH transcription factors, such as Tcf12 and E2A, bind to E-box enhancers of target genes in the shape of homodimers or heterodimers to regulate lymphocyte development and differentiation.^4^ Tcf12 and MyoD class of myogenic regulatory factors can also bind on the E-box sequences in the form of heterodimers in the promoter regions of muscle-specific genes to promote myogenesis.^5,6^

The Tcf12 gene encodes 2 isoforms, HEBAlt and HEBCan. HEBCan is expressed across almost all stages of T-cell development, while HEBAlt is expressed simply in the early phases of T cells development.^7^ HEBAlt regulates T cells fate and restricts myeloid fate in hematopoiesis.^8^ Targeted deletion of Tcf12 leads to about 10-fold reduction in the absolute number of thymocytes and this is owning to a developmental barrier from the double negative to double positive stage transition.^9–11^ A previous study depicted that the absolute number of CD150^+^ LSK (LSK: Lineage^−^ Sca-1^+^ c-Kit^+^) and CD150^−^ LSK cells kept unchanged in the bone marrow of HEB^f/f^Tie2Cre mice compared with the age-matched wild-type (WT) mice.^12^ While, there is still no report studying the effect of Tcf12 on the function of hematopoietic stem cells (HSCs).

In this study, we observed that targeted deletion of Tcf12 in hematopoietic cells increased the frequency and absolute number of HSCs, but the reconstitution capacity of HSCs is compromised. Further analysis of the secondary recipients reveal that Tcf12 is not essential for the self-renewal of HSCs. The proliferation of Tcf12^−/−^ HSC pool might due to a compensation for the differentiation defect of lymphoid-primed multipotent progenitor.

## 2 RESULTS

### 2.1 Tcf12 ablation induces HSCs expansion and impairs lymphoid-primed multipotent progenitor regeneration at steady state

**Figure 1:**
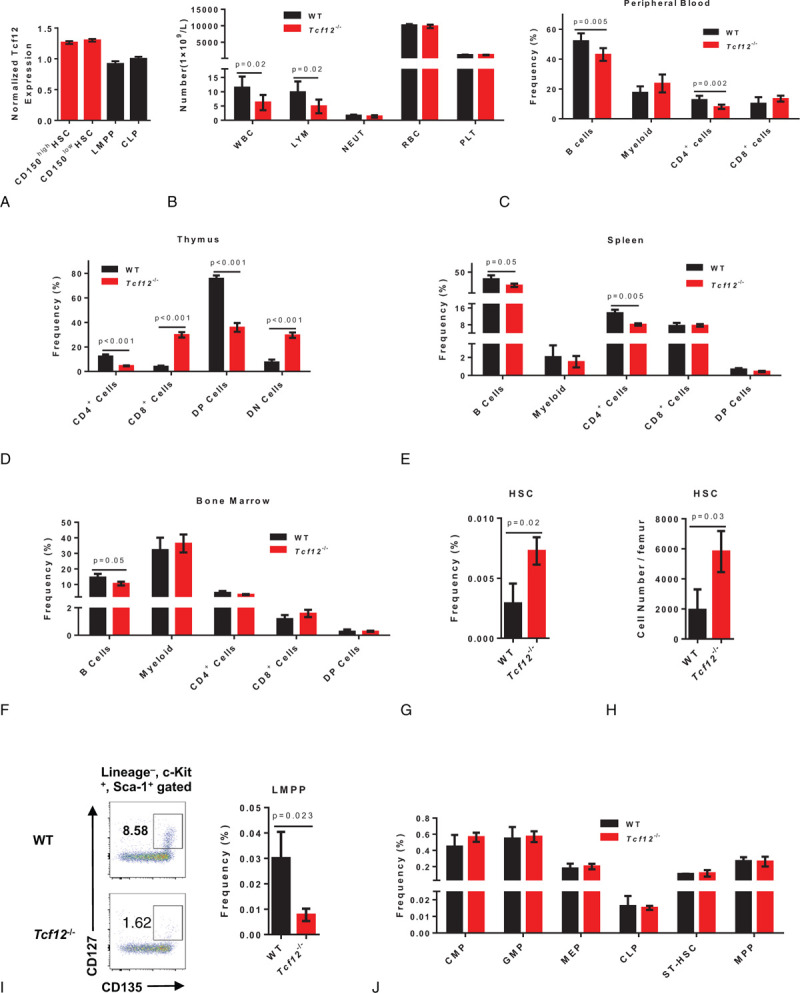
Tcf12 ablation induces HSCs expansion and impairs lymphoid-primed multipotent progenitor regeneration at steady state. (A) 1 × 10^4^ CD150^high^HSC (Lineage^–^ Sca1^+^ c-Kit^+^ CD150^high^ CD34^–^), CD150^low^HSC (Lineage^–^ Sca1^+^ c-Kit^+^ CD150^low^ CD34^–^), LMPP (Lineage^–^ Sca1^+^ c-Kit^+^ CD135^+^CD127^+^) and CLP (Lineage^–^ Sca1^low^ c-Kit^low^ CD135^+^ CD127^+^) cells freshly isolated from wild-type mice, respectively, were used for qRT-PCR analysis. The histogram displays the relative expression of Tcf12 in the indicated cells. (B) Seven 2 months old *Tcf12*^−/−^ mice (*Tcf12*^fl/fl^, *Vav-*icre^+^) and 7 age-matched WT mice (*Tcf12*^fl/fl^, *Vav*-icre^-^) were evaluated for white blood cell (WBC), lymphocyte (LYM), neutrophil (NEUT), red blood cell (RBC) and platelet (PLT) in the peripheral blood. The histogram shows the count of these cells between WT and *Tcf12*^−/−^ mice (Data are from 2 independent experiments). (C) The histogram depicts the frequency of B cells (B220^+^), myeloid (CD11b^+^), CD4^+^ cells and CD8^+^ cells in the peripheral blood of WT and *Tcf12*^−/−^ mice (n = 6 mice per group from 2 independent experiments). (D–F) Three 2 months old *Tcf12*^−/−^ mice and 3 age-matched WT mice were analyzed for B cells (B220^+^), myeloid (CD11b^+^), CD4^+^ cells, CD8^+^ cells, DP cells (CD4 and CD8 double positive cells) and DN cells (CD4 and CD8 double negative cells) in the thymus, spleen and bone marrow of WT and *Tcf12*^−/−^ mice. The histogram displays the frequency of the indicated cells in the thymus (D), spleen (E) and bone marrow (F) of WT and *Tcf12*^−/−^ mice (Data are from 2 independent experiments). (G-H) The histograms show the frequency (G) and absolute number (H) of HSC (Lineage^–^ Sca1^+^ c-Kit^+^ CD150^+^ CD34^–^) in WT and *Tcf12*^−/−^ mice (n = 3 mice per group from 2 independent experiments). (I) Representative dot plots from flow cytometry and the histogram exhibits the frequency of LMPP (Lineage^–^ Sca1^+^ c-Kit^+^ CD135^+^CD127^+^) in the bone marrow of WT and *Tcf12*^−/−^ mice (n = 3 mice/group from 2 independent experiments). (J) The histogram exhibiting the percentage of CMP (Lineage^–^ Sca1^–^ c-Kit^+^ CD16/32^–^ CD34^+^), GMP (Lineage^–^ Sca1^–^ c-Kit^+^ CD16/32^+^ CD34^+^), MEP (Lineage^–^ Sca1^–^ c-Kit^+^ CD16/32^–^ CD34^–^), CLP (Lineage^–^ Sca1^low^ c-Kit^low^ CD135^+^ CD127^+^), ST-HSC (Lineage^–^ Sca1^+^ c-Kit^+^ CD135^–^ CD34^+^) and MPP (Lineage^–^ Sca1^+^ c-Kit^+^ CD135^+^ CD34^+^) in the bone marrow of WT and *Tcf12*^−/−^ mice (n = 3 mice per group from 2 independent experiments).

Tcf12 is a master regulator of T cells and early thymic progenitors. However, we observed that the expression level of Tcf12 in HSCs (both Lineage^–^ Sca1^+^ c-Kit^+^ CD150^high^ CD34^–^ and Lineage^–^ Sca1^+^ c-Kit^+^ CD150^low^ CD34^–^ cells) is more abundant than that in LMPP (lymphoid-primed multipotent progenitor) and CLP (common lymphoid progenitor) (Fig. 1A), implicating a potential role of Tcf12 in modulating HSCs and hematopoiesis. While the expression of Tcf12 shows no differences between lymphoid-biased HSCs (Lineage^–^ Sca1^+^ c-Kit^+^ CD150^low^ CD34^–^) and myeloid-biased HSCs (Lineage^–^ Sca1^+^ c-Kit^+^ CD150^high^ CD34^–^) (Fig. 1A).

To elucidate the function of Tcf12 in hematopoiesis, *Tcf12*^fl/fl^ mice were crossed with *Vav-*iCre mice to generate *Vav-*iCre;*Tcf12*^fl/fl^ mice, wherein Tcf12 deletion occurs primarily in hematopoietic cells (hereafter called *Tcf12*^−/−^). The numbers of white blood cells (WBC) and lymphocytes (LYM) exhibited a significant decrease in the peripheral blood of *Tcf12*^−/−^ mice at 2 months compared with age-matched WT mice, while neutrophil (NEUT), red blood cell (RBC) and platelet (PLT) counts remained static (Fig. 1B). Meanwhile, we also observed the percentage of B cells and CD4^+^ cells declined significantly in the peripheral blood of *Tcf12*^−/−^, but myeloid and CD8^+^ cells kept unchanged (Fig. 1C). Moreover, the frequency and absolute number of CD4^+^ cells and double positive T cells (DP cells, CD4^+^CD8^+^) decreased significantly in the thymus of *Tcf12*^−/−^ mice, while CD8^+^ cells and double negative T cells (DN cells, CD4^-^CD8^-^) displayed a reverse trend (Figs. 1D, S1A and S1B) which is consistent with previous reports.^9–11^ Lineage cells in the spleen of *Tcf12*^−/−^ and WT mice were also evaluated, and the results showed the percentage and absolute number of B cells and CD4^+^ cells declined significantly in *Tcf12*^−/−^ mice while myeloid, CD8^+^ cells and DP cells holds static (Fig. 1E, S1C and S1D). Furthermore, only the percentage and absolute number of B cells were decreased significantly in the bone marrow of *Tcf12*^−/−^ mice compare to age-matched WT mice (Fig. 1F, S1E and S1F). The frequency and absolute number of HSCs (Lineage^–^ Sca1^+^ c-Kit^+^ CD150^+^ CD34^–^) in *Tcf12*^−/−^ mice were significantly larger than WT mice (Fig. 1G, 1H and S1G). Meanwhile, the percentage of both lymphoid-biased HSCs and myeloid-biased HSCs increased significantly in *Tcf12*^−/−^ mice, which indicates the differentiation block of lineage cells may not take place from myeloid- and lymphoid-biased HSCs (Fig. S1I). However, LMPP of *Tcf12*^−/−^ mice reduced to 30% of WT mice (Fig. 1I), but the other progenitor cells remain unchanged (Fig. 1J, S1G and S1H). These data suggest that Tcf12 deletion impairs lymphoid-primed multipotent progenitor regeneration at steady state.

### 2.2 Tcf12 loss impairs HSC differentiation upon transplantation

**Figure 2:**
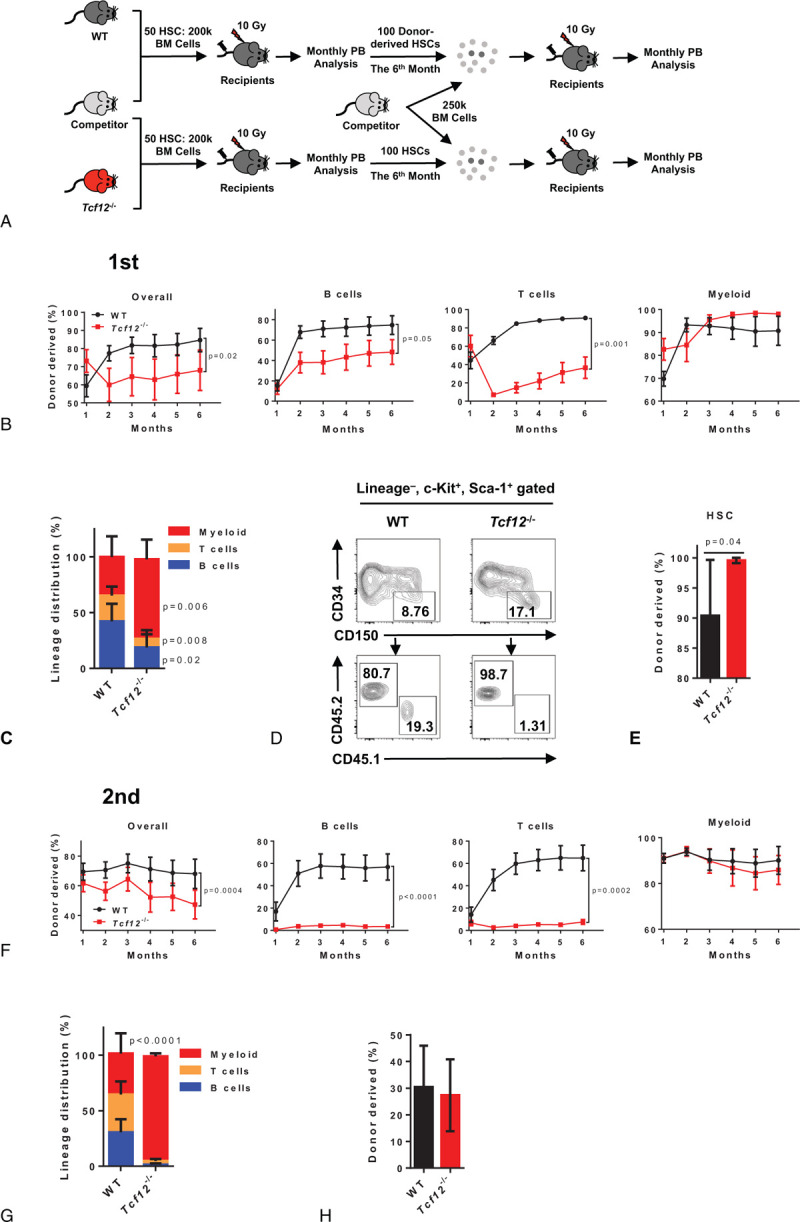
Tcf12 loss impairs HSC differentiation upon transplantation. (A) The schematic diagram depicts the experimental design to evaluate the function of *Tcf12*^−/−^ HSCs. 50 WT or *Tcf12*^−/−^ HSCs (CD45.2) together with 2 × 10^5^ competitor cells (CD45.1) were transplanted into lethally irradiated recipients (CD45.1/2). Chimerism in the peripheral blood was analyzed monthly until the sixth month when all recipients were sacrificed and 100 donor-derived HSCs were sorted from the indicated mice along with 2.5 × 10^5^ competitor cells for the secondary competitive transplants. Chimerism in the peripheral blood was evaluated monthly until the sixth month. (B) These line plots display the frequency of donor-derived cells in overall cells (CD45.2^+^), B cells (B220^+^), T cells (CD3^+^) and myeloid (CD11b^+^) at the indicated time points in the peripheral blood of WT and *Tcf12*^−/−^ primary recipient mice (n = 6 mice per group from 2 independent experiments). (C) The histogram depicts the lineage distribution of myeloid, T cells and B cells among donor-derived cells in the peripheral blood of the indicated primary recipients at the 6^th^ month after transplantation (n = 6 mice per group from 2 independent experiments). (D-E) Representative plots from flow cytometry (D) and the histogram (E) shows the percentage of donor-derived HSC (Lineage^–^ Sca1^+^ c-Kit^+^ CD34^–^CD150^+^) in the indicated primary recipients at the 6^th^ month after transplantation (n = 6 mice/group from 2 independent experiments). (F) The line graphs display the percentage of donor-derived cells in overall cells (CD45.2^+^), B cells (B220^+^), T cells (CD3^+^) and myeloid (CD11b^+^) at the indicated time points in the peripheral blood of WT and *Tcf12*^−/−^ secondary recipient mice (n = 7 mice per group from 2 independent experiments). (G) The histogram exhibits the lineage distribution of the indicated cells among donor-derived cells in the peripheral blood of WT and *Tcf12*^−/−^ secondary recipients at the 6^th^ month after transplantation (n = 7 mice per group from 2 independent experiments). (H) The histogram depicts the percentage of donor-derived HSC (Lineage^–^ Sca1^+^ c-Kit^+^ CD34^–^) in the indicated secondary recipients at the 6^th^ month after transplantation (n = 7 mice per group from 2 independent experiments).

To further evaluate the role of Tcf12 in HSC function, 50 freshly purified HSCs (Lineage^–^ Sca1^+^ c-Kit^+^ CD150^+^ CD34^–^) from either *Tcf12*^−/−^ mice or WT mice were transplanted into lethally irradiated recipients together with 2 × 10^5^ competitor cells, and the chimera was evaluated every month until the 6^th^ month (Fig. 2A). The result displays that the reconstitution capacity of Tcf12^−/−^ HSCs is significantly lower than that of WT HSCs (84.6% ± 15.7% vs 68.0% ± 27.2%), and this effect mainly stems from the differentiation defect of B and T cells, but not myeloid (Fig. 2B and S1A). Meanwhile, *Tcf12*^−/−^ HSCs display differentiation bias towards myeloid lineage at the expense of B cells and T cells (Fig. 2C and S2B). The primary recipients were sacrificed at 6 months after transplantation and the donor-derived HSCs in the bone marrow were analyzed. The result shows that the frequency of donor-derived *Tcf12*^−/−^ HSCs reached almost 100% which is significantly higher than that (about 90%) of the WT control (Fig. 2D, E), indicating that deletion of Tcf12 may enhance the proliferation rate or self-renewal capacity of HSCs. Furthermore, both lymphoid-biased and myeloid-biased *Tcf12*^−/−^ HSCs displayed significant growth advantage compared to corresponding WT HSCs (Fig. S2C). Then, 100 *Tcf12*^−/−^ or WT HSCs from the primary recipient mice were freshly purified and transplanted into lethally irradiated recipients along with 2.5 × 10^5^ freshly isolated competitor cells (Fig. 2A). The results exhibit that the engraftment of *Tcf12*^−/−^ HSCs in secondary recipients was significantly lower than that of WT control (Fig. 2F). Meanwhile, the capacity of *Tcf12*^−/−^ HSCs differentiation into B cells and T cells was almost completely lost, while no effect was observed in myeloid lineage differentiation (Fig. 2F, G). However, Tcf12^−/−^-derived HSCs display no difference compared to WT control (Fig. 2H), which indicates that the higher frequency of donor-derived HSCs in the *Tcf12*^−/−^ primary recipients may due to increase the proliferation of *Tcf12*^−/−^ HSCs rather than enhancing its self-renewal ability.

### 2.3 *Tcf12* deletion in HSCs leads to down-regulation of HSC differentiation genes

**Figure 3:**
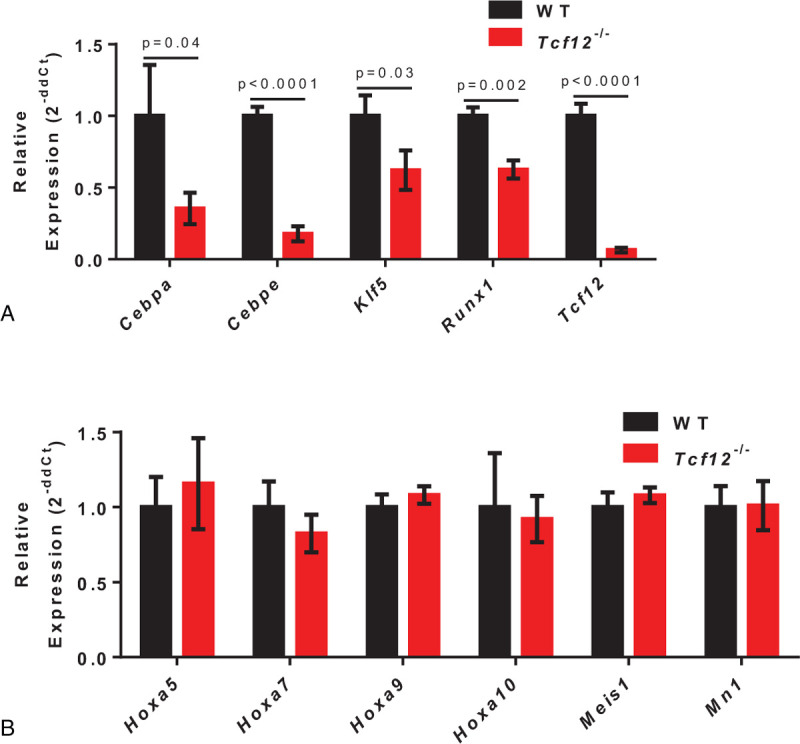
*Tcf12* deletion in HSCs leads to down-regulation of HSC differentiation genes. (A, B) 5000 HSCs freshly isolated from *Tcf12*^−/−^ or WT mice were used for qRT-PCR analysis. The histograms showing the relative expression of differentiation-related genes (A) and HSC multipotency genes (B) in the indicated cells.

Given our data above show that *Tcf12* ablation impairs HSC differentiation but not HSC self-renewal, we then set out to evaluate the expression of related genes in *Tcf12*^−/−^ and WT HSCs by qRT-PCR. Consistent with our aforementioned hypothesis, the expression of several genes that regulate HSC differentiation were significantly reduced in *Tcf12*^−/−^ HSCs (Lineage^–^ Sca1^+^ c-Kit^+^ CD150^+^ CD34^–^), including *Cebpa*, *Cebpe*, *Klf5* and *Runx1* (Fig. 3A). However, the expression of HSC self-renewal related genes, such as *Hoxa5*, *Hoxa7*, *Hoxa9*, *Hoxa10* and *Mn1*, did not show any difference in HSCs of *Tcf12*^−/−^ and WT control mice (Fig. 3B). Together, all data above suggest that *Tcf12* regulates HSC differentiation instead of self-renewal.

## 3 DISCUSSION

At steady state, the frequency and absolute number of HSCs in *Tcf12*^−/−^ mice increase significantly, while another E protein named E2A which is indispensable for the maintenance of HSC pool shows a reduced numbers of HSCs in *E2A*-null mice.^14^ Therefore, it is important to note whether Tcf12 deletion enhances the self-renewal ability of HSCs or merely increases proliferation of HSCs. From the competitive HSC transplantation assay, we found that *Tcf12*^−/−^ HSCs exhibited a decreased contribution to T cells and B cells reconstitution in the primary recipients and almost failed to reconstitute these 2 cell lineage in the secondary recipients, whereas myeloid reconstitution was unaffected. Previous report showed that Tcf12 is dispensable for the establishment of the B-cell lineage but pro-B cells,^15^ while our data shows that B cells reconstitution in the secondary recipients was severely damaged. This might due to a lack of evaluating *Tcf12*^−/−^ HSC function by serial transplantation assay.

Six months after the primary transplantation, donor-derived HSCs increase significantly in spite of the decreased reconstitution ability in *Tcf12*^−/−^ recipients compared with WT recipients. These increased HSCs might stem from a proliferation of HSC pool at the expense of the differentiation defect of B and T cells, and this may also be the reason why the frequency and absolute number of HSCs in *Tcf12*^−/−^ mice increase significantly at steady state. However, there is no difference in donor-derived HSCs between the secondary recipients of the WT and *Tcf12*^−/−^ mice. We speculate that this might be due to *Tcf12*^−/−^ HSCs confronted with a strong replication stress under serial transplantation and the replication stress dominate the proliferation effect of HSCs. These data indicate Tcf12 loss in HSCs promotes proliferation of HSCs rather than enhancing self-renewal.

## 4 MATERIAL AND METHODS

### 4.1 Mice

*Tcf12*^fl/fl^ mice were provided by Dr. Yuan Zhuang from Duke University and were generated as described.^13^*Vav*-iCre mice were obtained from the Jackson Lab. Tcf12^fl/fl^ mice were crossed with Vav-iCre mice to generate Tcf12 knockout mice, wherein Tcf12 deletion occurs mainly in hematopoietic cells (hereafter named *Tcf12*^−/−^ mice or *Tcf12*^−/−^ HSCs). In all experiments (unless otherwise specified), mice were homozygous for *Tcf12* allele and heterozygous for *Vav*-iCre allele. *Tcf12*^fl/fl^*Vav*-iCre^+^ (*Tcf12*^−/−^) mice were used for the experiment and *Tcf12*^fl/fl^*Vav*-iCre^-^ mice were applied for the WT control. All mice were kept in specific-pathogen-free (SPF), AAALAC-accredited animal care facilities at the Laboratory Animal Research Center, Tsinghua University and all procedures were approved by Institutional Animal Care and Use Committee of Tsinghua University.

### 4.2 Flow cytometric analysis and cell sorting

All cells freshly isolated from mice were suspended in HBSS buffer supplemented with 2% fetal bovine serum, 1% penicillin/streptomycin and 1% HEPES, and then stained with the indicated fluorochrome-labeled antibodies. Flow cytometric analysis was performed with a BD LSRFortessa SORP flow cytometer (BD Biosciences) and data were analyzed using FlowJo™ Software (Becton, Dickinson and Company). BD Influx (BD Biosciences) was applied for cell sorting and the desired fractions were sorted into the indicated buffer. Non-lysed bone marrow cells were used for analysis of HSC and progenitor cells (antibodies including Lin-biotin cocktails, Streptavidin APC/Cy7, Sca-1 PE/Cy7, c-Kit APC, CD34 AF700, CD150 PE, CD135 PE-CF594, CD16/32 FITC and CD127 BV421). Mature lineage cells from peripheral blood were pretreated by ACK buffer (KHCO_3_ 10 mM, NH_4_Cl 150 mM, Na_2_EDTA 0.1 mM, adjust the pH to 7.2–7.4) and stained with indicated antibodies (antibodies containing CD11b Percpcy5.5, B220 PB, CD3 APC, CD45.1 FITC and CD45.2 PE) before being subjected to flow cytometer for analysis.

### 4.3 Competitive HSC transplantation

For the primary competitive HSC transplantation assay, 50 freshly isolated HSCs from WT or *Tcf12*^−/−^ mice (CD45.2, C57BL/6J) along with 2 × 10^5^ WT (CD45.1, C57BL/6J) whole bone marrow competitor cells were injected intravenously (i.v.) into lethally irradiated (10 Gy) WT recipient mice (CD45.1/2, F1 generated by mating CD45.1 with CD45.2 mice, C57BL/6J). Donor-derived chimaerism (including B cells, T cells and myeloid) in peripheral blood of recipients were analyzed monthly interval. After 6 months, all recipient mice were sacrificed for donor-derived HSC analysis and the secondary competitive HSC transplantation. For the secondary competitive HSC transplantation assay, 100 freshly isolated donor-derived HSCs (CD45.2) from WT or *Tcf12*^−/−^ recipient mice were transplanted into lethally irradiated (10 Gy) recipient mice (CD45.1/2) together with 2.5 × 10^5^ WT whole bone marrow competitor cells (CD45.1). The chimera in recipients was evaluated for 6 months before all mice were sacrificed for donor-derived HSC analysis.

### 4.4 Hematological cell counts

The whole blood bled from the tail of mice was subjected to Auto Hematology Analyzer BC-5000 (MINDRAY) for hematologic parameters analysis. Bone marrow cells freshly isolated from mice were suspended in the indicated buffer on ice before analyzing by Vi-CELL Cell Counter (Beckman).

### 4.5 Quantitative real-time PCR

A total of 5000 HSCs freshly sorted by BD Influx (BD Biosciences) from bone marrow of 2 months WT or *Tcf12*^−/−^ mice were lysed in TRIzol (Invitrogen) for total RNA extraction according to the manufacturer's instructions. The cDNA was synthesized from the indicated RNA by PrimeScript RT reagent Kit (Takara, Cat # RR047A). The cDNA obtained above was mixed with the indicated primers and analyzed using PowerUp™ SYBR™ Green mix (Applied Biosystems, Cat # A25780) according to the manufacturer's instructions on a QuantStudio-3 Real-time PCR System (Applied Biosystems). The detailed primer information is listed in Table S1.

### 4.6 Statistical analysis

Two-tailed unpaired Student's *t* test was applied for analyzing experiment data after testing for normal distribution. All histogram and line graph were plotted by GraphPad Prism 6 software, and *P* < .05 was regarded as significant for all tests. All data are depicted as mean ± SD.

## Supplementary Material

Supplemental Digital Content
