# Supplementary material for: Tcf12 balances the reconstitution and differentiation capacity of hematopoietic stem cell
Source: Blood Sci. 2020 Dec 1;3(1):14–9. doi: 10.1097/BS9.0000000000000059 (PMC8974954; doi:10.1097/BS9.0000000000000059)
Supplement: Supplemental Digital Content [file bls-3-14-s001.pdf]

Fig. S1

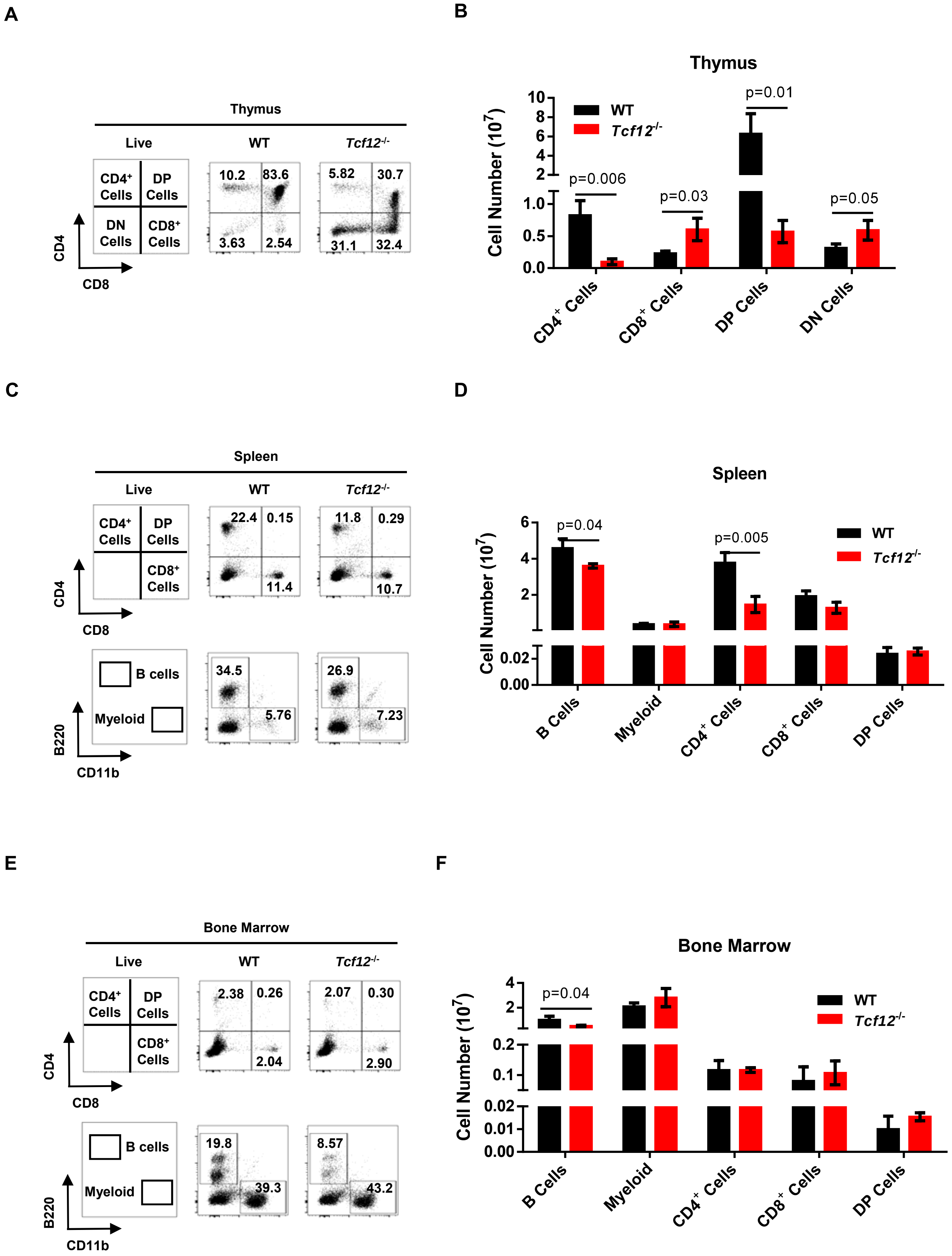

A
Thymus
Live
WT
Tcf12-/-
CD4+ Cells
DP Cells
DN Cells
CD8+ Cells
10.2
83.6
3.63
2.54
5.82
30.7
31.1
32.4
CD4
CD8
B
Thymus
Cell Number (107)
WT
Tcf12-/-
p=0.006
p=0.03
p=0.01
p=0.05
CD4+ Cells
CD8+ Cells
DP Cells
DN Cells
C
Spleen
Live
WT
Tcf12-/-
CD4+ Cells
DP Cells
CD8+ Cells
22.4
0.15
11.4
11.8
0.29
10.7
CD4
CD8
B cells
Myeloid
34.5
5.76
26.9
7.23
B220
CD11b
D
Spleen
Cell Number (107)
p=0.04
p=0.005
WT
Tcf12-/-
B Cells
Myeloid
CD4+ Cells
CD8+ Cells
DP Cells
E
Bone Marrow
Live
WT
Tcf12-/-
CD4+ Cells
DP Cells
CD8+ Cells
2.38
0.26
2.04
2.07
0.30
2.90
CD4
CD8
B cells
Myeloid
19.8
39.3
8.57
43.2
B220
CD11b
F
Bone Marrow
Cell Number (107)
p=0.04
WT
Tcf12-/-
B Cells
Myeloid
CD4+ Cells
CD8+ Cells
DP Cells

Fig. S1

G

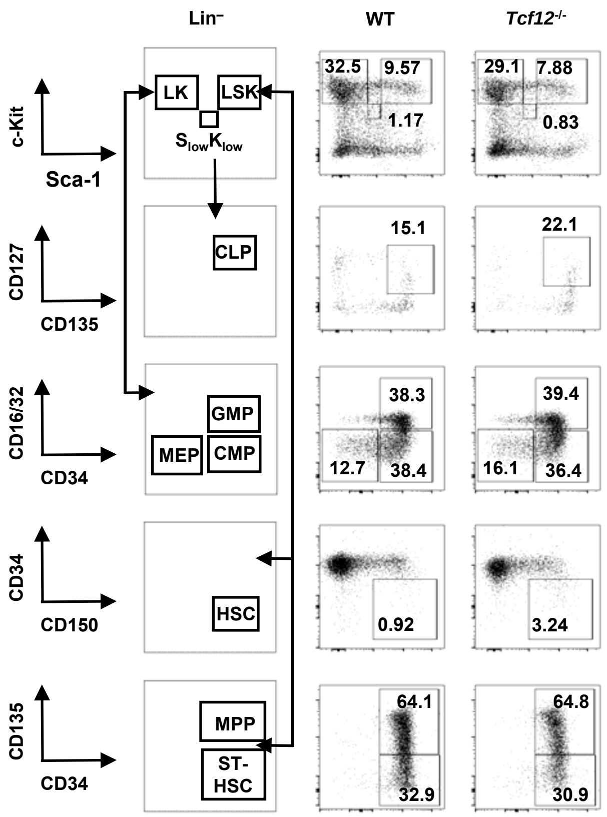
Lin⁻
WT
Tcf12-/-
c-Kit
Sca-1
LK
LSK
SlowKlow
32.5
9.57
1.17
29.1
7.88
0.83
CD127
CD135
CLP
15.1
22.1
CD16/32
CD34
GMP
MEP
CMP
38.3
12.7
38.4
39.4
16.1
36.4
CD34
CD150
HSC
0.92
3.24
CD135
CD34
MPP
ST-HSC
64.1
32.9
64.8
30.9

H

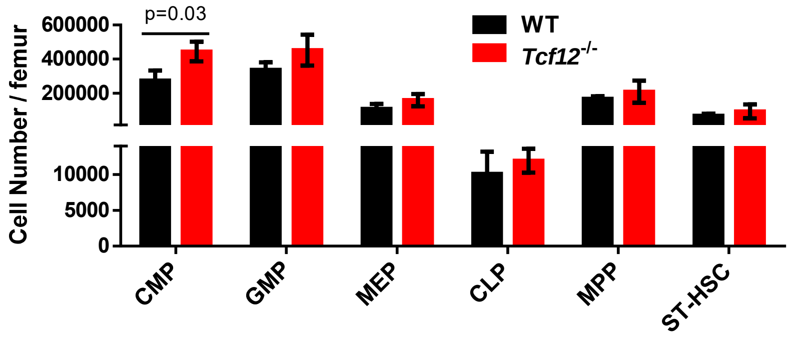
p=0.03
WT
Tcf12-/-
Cell Number / femur
600000
400000
200000
10000
5000
0
CMP
GMP
MEP
CLP
MPP
ST-HSC

I

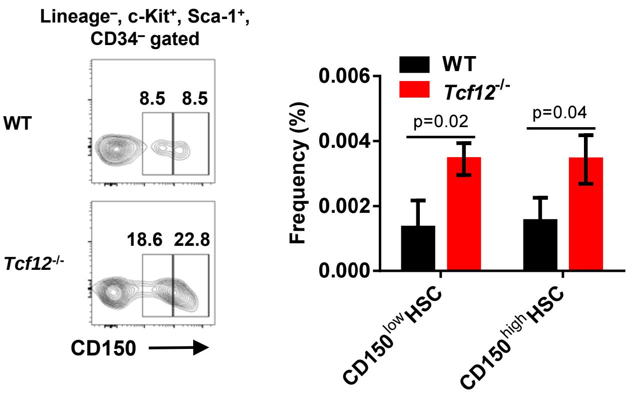
Lineage⁻, c-Kit⁺, Sca-1⁺, CD34⁻ gated
WT
8.5
8.5
Tcf12-/-
18.6
22.8
CD150
WT
Tcf12-/-
Frequency (%)
0.006
0.004
0.002
0.000
p=0.02
p=0.04
CD150low HSC
CD150high HSC

Fig. S2

A

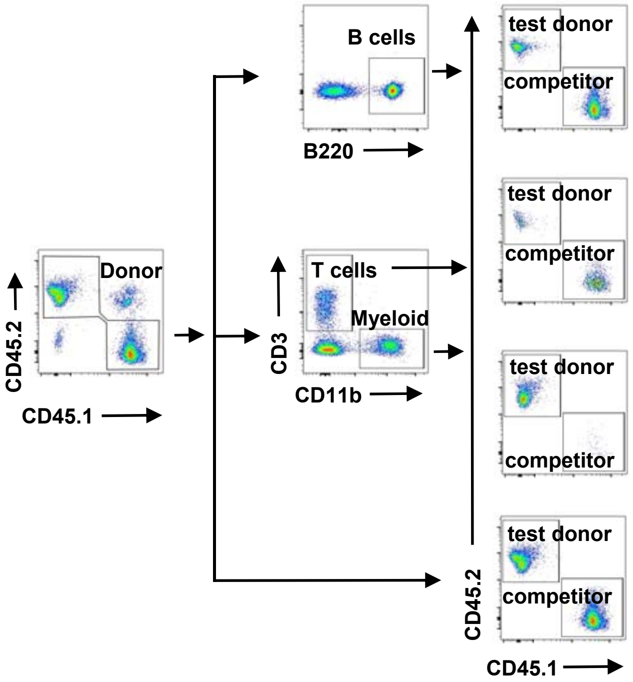

Donor
CD45.2
CD45.1
B cells
B220
T cells
Myeloid
CD3
CD11b
test donor
competitor
test donor
competitor
test donor
competitor
test donor
competitor
CD45.2
CD45.1

B

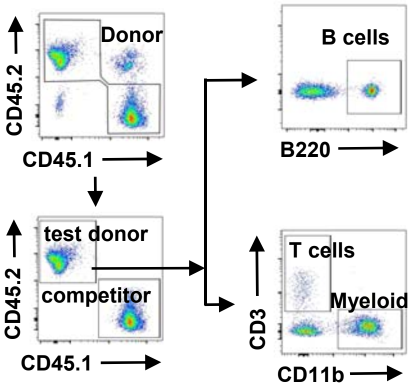

Donor
CD45.2
CD45.1
test donor
competitor
CD45.2
CD45.1
B cells
B220
T cells
Myeloid
CD3
CD11b

C

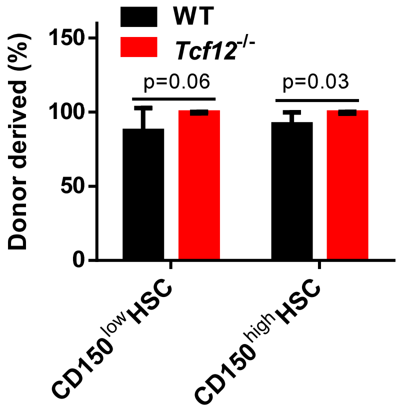

WT
Tcf12-/-
Donor derived (%)
150
100
50
0
p=0.06
p=0.03
CD150lowHSC
CD150highHSC

Figure S1

(A-B) Representative plots display the gating strategies for $CD4^+$ cells, $CD8^+$ cells, DP cells (double positive cells, $CD4^+CD8^+$) and DN cells (double negative cells, $CD4^-CD8^-$) analysis in the thymus of WT and *Tcf12*$^{-/-}$ mice (A), and the histogram shows the absolute cell number of the indicated cell population (B) (n = 3 mice per group from two independent experiments, data are shown as mean ± SD).

(C-D) Representative dot plots exhibiting the gating strategies for quantifying the percentage of lineage cells ($CD4^+$ cells, $CD8^+$ cells, DP cells, myeloid and B cells) in the spleen of WT and *Tcf12*$^{-/-}$ mice (C) and the histogram depicts the absolute cell number of the each cell populations (D) (n = 3 mice/group from two independent experiments, data are shown as mean ± SD).

(E-F) Representative flow cytometry plots showing the gating strategies for lineage cells ($CD4^+$ cells, $CD8^+$ cells, DP cells, myeloid and B cells) analysis in the bone marrow of WT and *Tcf12*$^{-/-}$ mice (E) and the histogram displays the absolute cell number of the indicated cell populations (F) (n = 3 mice per group from two independent experiments, data are shown as mean ± SD).

(G-H) Representative plots depict the gating strategies for HSC and progenitor cells (CMP, GMP, MEP, CLP, MPP and ST-HSC) analysis in the bone marrow of WT and *Tcf12*$^{-/-}$ mice (G) and the histogram exhibits the absolute cell number of the progenitor cells in the indicated mice (H) (n = 3 mice per group from two independent experiments, data are shown as mean ± SD).

(I) Representative plots from flow cytometry and the histogram shows the frequency of $CD150^{high}$HSC and $CD150^{low}$HSC in the bone marrow of WT and *Tcf12*$^{-/-}$ mice (n = 3 mice per group from 2 independent experiments).

Figure S2

(A-B) Representative flow cytometry plots depicting the gating strategies for quantifying the frequency of test donor-derived lineage cells (B cells, T cells and

myeloid) (A) and lineage distribution of B cells, myeloid, T cells within test donor-derived cells (B) in the peripheral blood of WT and *Tcf12*$^{-/-}$ recipient mice in the competitive transplantation assay.

(C) The histogram exhibits the frequency of donor-derived CD150^{high}HSC and CD150^{low}HSC in the indicated primary recipients at the 6th month after transplantation (n = 6 mice per group from 2 independent experiments).

Table S1. Primers for Genotyping and qRT-PCR

Oligonucleotides				
**Gene**	**Forward**	**Reverse**	**Application**	**IDENTIFIER**
Vav-iCre	5’-AGATGCCAGGACATCAGGAACCTG-3’	5’-ATCAGCCACACCAGACACAGAGATC-3’	Genotyping	N/A
Tcf12	5’-CCGTGGCAGTCATCCTTAGT-3’	5’-TAACTGCCCAGCACAGAAGA-3’	Genotyping	N/A
Cebpa	5’-TTGTTTGGCTTTATCTCGGC-3’	5’-CCAAGAAGTCGGTGGACAAG-3’	qRT-PCR	N/A
Cebpe	5’-ATCGACCTCTCCGCCTACAT-3’	5’-TTAAGGCTTCGGGCTTCAGG-3’	qRT-PCR	N/A
Klf5	5’-CCGGAGACGATCTGAAACACG-3’	5’-GTTGATGCTGTAAGGTATGCCT-3’	qRT-PCR	N/A
Runx1	5’-CCGAGAACCCCGAAGACATC-3’	5’-GCAGTGGAGTGGTTCAAGGA-3’	qRT-PCR	N/A
Gapdh	5’-AGGTCGGTGTGAACGGATTTG-3’	5’-TGTAGACCATGTAGTTGAGGTCA-3’	qRT-PCR	N/A
Actin	5’-GTGACGTTGACATCCGTAAAGA-3’	5’-GCCGGACTCATCGTACTCC-3’	qRT-PCR	N/A
Tcf12	5’-GGAAAGGCGGATGGCTAACA-3’	5’-ACGGCCTGATGAAGAATGAGA-3’	qRT-PCR	N/A
Hoxa5	5’-TACGGCTACAATGGCATGGAT-3’	5’-CCGCTGGAGTTGCTTAGGG-3’	qRT-PCR	N/A
Hoxa7	5’-CGTTCCGGGCTTATACAATGT-3’	5’-CTCGTCCGTCTTGTCGCAG-3’	qRT-PCR	N/A
Hoxa9	5’-GTCCAAGGCGACGGTGTTT-3’	5’-CCGACAGCGGTTCAGGTTTA-3’	qRT-PCR	N/A
Hoxa10	5’-CTCGCCCATAGACCTGTGG-3’	5’-GTTCTGCGCGAAAGAGCAC-3’	qRT-PCR	N/A
Meis1	5’-TACCCGCACACAGCTCATAC-3’	5’-CATTGAATGACTCTGACGAGCA-3’	qRT-PCR	N/A
Mn1	5’-GGACTTCCATAGTTCGGGCA-3’	5’-TGAGAATCCGAGCCGCTAGA-3’	qRT-PCR	N/A
